# Physicochemical characteristics of the Dombrovska pit lake (Ukraine) formed in an opencast potassium salt mine and the genome response of *Chironomus salinarius* Kieffer (Chironomidae, Diptera) to these conditions

**DOI:** 10.1007/s11356-020-10465-0

**Published:** 2020-08-19

**Authors:** Paraskeva Michailova, Ewa Szarek-Gwiazda, Andrzej Kownacki

**Affiliations:** 1 Institute of Biodiversity and Ecosystem Research, Bulgarian Academy of Sciences, 1 Tzar Osvoboditel Boulv., 1000 Sofia, Bulgaria; 2 Institute of Nature Conservation, Polish Academy of Sciences, Adama Mickiewicza 33, 31-120 Krakow, Poland; 3 Polish Hydrobiological Society, Krakow Branch, Adama Mickiewicza 33, 31-120 Krakow, Poland

**Keywords:** Dombrovska lake, Saline water, Benthic macro-invertebrate, *Chironomus salinarius*, Aberrations in polytene chromosomes

## Abstract

This study focuses on the Dombrovska pit lake, near the city of Kalush in Ukraine, which is a former potassium salt mine filled with brine and freshwater. The water level is still increasing and as a result the salinity is decreasing. We analyzed the benthic fauna communities and the genome instability by assessing the rearrangements in the polytene chromosomes of *Chironomus salinarius* and the physicochemical parameters of the near-bottom water (pH, conductivity, mineralization, major ions, NO_3_^−^, NH_4_^+^, metals Cd, Pb, Cu, Mn, and Fe) and sediment (pH, organic matter and metals Cd, Pb, Cu, Zn, Mn, and Fe) at four sites. The water mineralization ranged from 17.3 to 26.2 g dm^−3^ which are classified as mesohaline and polyhaline waters, respectively. The biodiversity of the benthic fauna was low, and the dominant species was *C. salinarius.* The density of *C. salinarius* varied spatially and changed from 637 ind./m^2^ at a depth of 5 m to 8167 ind./m^2^ at a depth of 2.5 m. The genome instability was analyzed by examining the structural and functional changes in the salivary gland chromosomes of *C. salinarius*. The exposure of *C. salinarius* damaged the chromosomes and the activities of key structures, such as the Balbiani ring and nucleolar organizer, were partially or completely suppressed.

## Introduction

The chemistry of water reservoirs that are created in former mine excavations usually lead to unique living conditions for biota. The Dombrovska pit lake near the city of Kalush in Ukraine is one of the most saline inland water bodies in the world (Zurek et al. 2018). It formed in a former opencast potassium salt mine, which operated from 1967 to 2005 and was closed between 2005 and 2008. Then, the mine excavation started filling with highly mineralized quaternary water, rainwater, and groundwater seepage (Gajdin et al. 2014). An annual inflow of ~ 2 million m^3^ of water results in the water level increasing by ~ 4 m every year (Dolin et al. 2010). Mineralization of the surface layer of water (0–5 m) was extremely high (165–301 g dm^−3^) in the northern part of the pit lake in 2009 but over time it decreased to 20–105 g dm^−3^ in 2014 (Gajdin et al. 2014). In terms of water mineralization, in 2015 the deepest central part of the pit lake had two distinct layers: the surface layer (0–5 m) was well oxygenated with mineralization of 50–134 g dm^−3^, and the underlying layer (up to a depth of 85 m) was poorly oxygenated with a mineralization 179–420 g dm^−3^ (Zurek et al. 2018). It is known that the salinity is the main parameter governing the biological biodiversity (Mirabdullayev et al. 2004; Zinchenko et al. 2019). The high levels of water mineralization of the Dombrovska pit lake creates unsuitable conditions for most biota, thus regarding zooplankton only three living taxa have been found: the rotifer *Brachionus plicatilis* and the ciliates *Paradileptus elephantinus* and *Tindinnidium*. In the littoral part of the pit lake diatoms that are resistant to high salinity, such as *Nitzschia pusilla*, *Halamphora borealis*, *H. tenerrima*, and *H. acutiuscula,* have been found. The pit lake is not yet completely filled with water and the final state of water quality has not been reached (Zurek et al. 2018).

A preliminary study of benthic fauna in 2018 showed that the *Chironomus* (*Chironomus*) *salinarius* Kieffer were dominant in the Dombrovska pit lake. *C. salinarius* inhabit aquatic ecosystems with a wide salinity spectrum and tolerate important variations in salinity (Arias and Drake 1994; Cartier et al. 2011; Drake and Arias 1995; Gascon et al. 2007; Hiebaum 2007; Michailova 1973, 1974; Ponti et al. 2007; Zinchenko et al. 2019; Zorina et al. 2014). This species appears in Palearctic regions and is very abundant in saline waters, as well as mesotrophic and eutrophic rivers and lakes (Cartier et al. 2011; Zorina et al. 2014). Moreover, this species is multivoltine, with a varying number of generations per year (Cartier et al. 2011; Drake and Arias 1995; Ferrarese et al. 2018; Koskinen 1968). It is also a dominant species in stressed habitat types (temporary waters, low sand proportion, and high salinity) (Gascon et al. 2007). The genome characteristics of *C. salinarius* are well described by Grinchuk (1979, 1984) for Ukraine, by Istomina et al. (2012), Kiknadze et al. (2016) and Zorina et al. (2014) for Russian populations, and by Michailova (1973, 1974, 1989) for Bulgarian population as well as by Keyl and Keyl (1959) and Keyl (1962) for the German population.

The aim of the study is to determine the physicochemical parameters of the Dombrovska pit lake and the communities of the benthic fauna and to track the response of the *C. salinarius* genome to stress conditions of decreasing salinity and increasing water levels.

## Materials and methods

### Sample area

The Dombrovska pit lake (49° 01′ 34.18″ N, 24° 19′ 24.74″ E) is located near the city of Kalush in Ukraine. In November 2015, the pit lake was 1770-m long, 260–450-m wide, and 85-m deep (Zurek et al. 2018). The bottom of the pit lake is covered by loams with a thickness of 2.5–6 m (Gajdin et al. 2014). A detailed description of the pit lake is given by Zurek et al. (2018). There are no fish in the pit lake; therefore, fish predation on benthic fauna can be omitted. The pit lake was not overgrown by macrophytes.

### Sample sites

**Fig 1:**
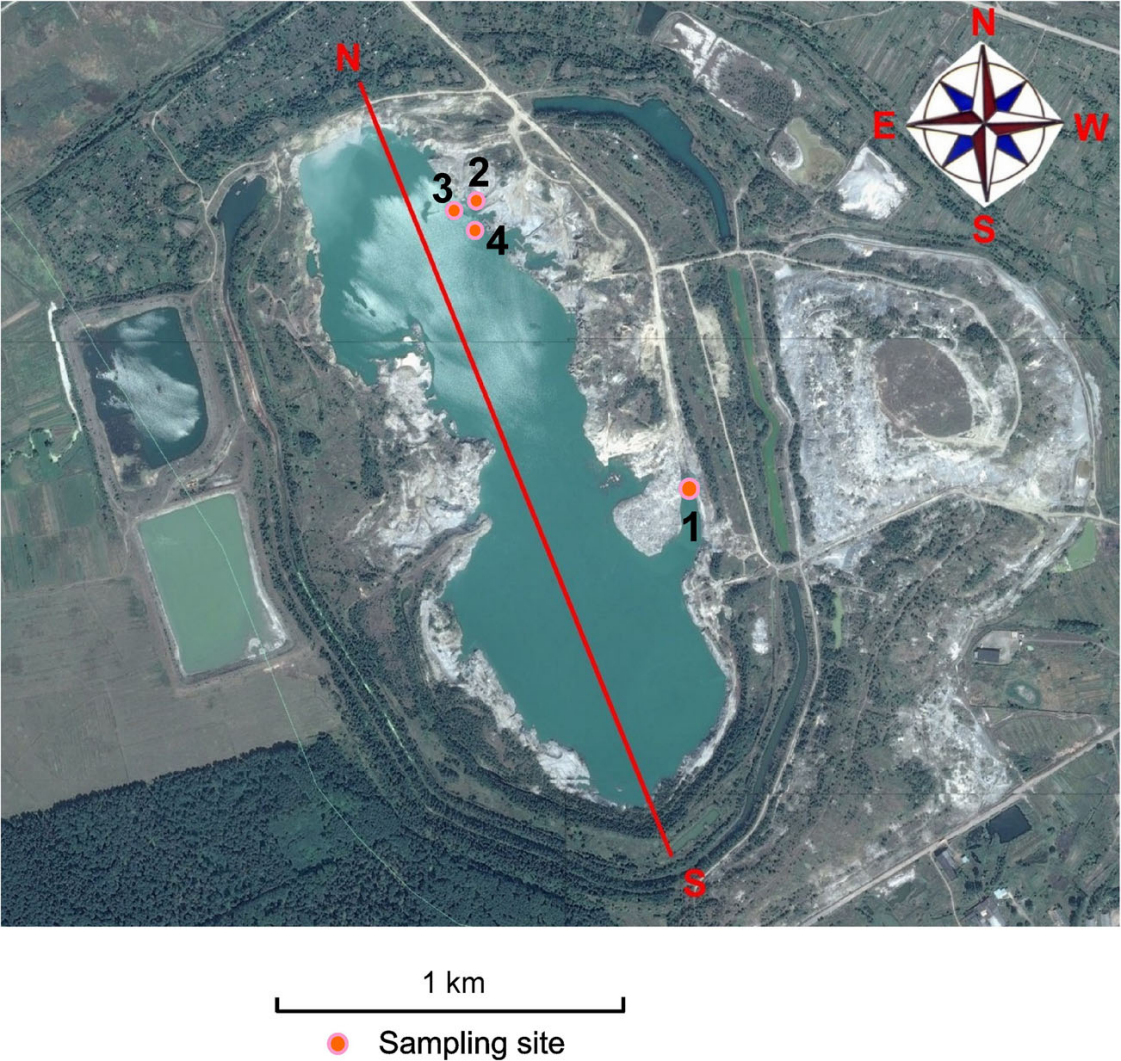
Location of the sampling sites at the Dombrovska pit lake, Ukraine

Samples of the near-bottom water and sediment for physicochemical analysis together with zoobenthos samples were collected from four sites from the western part of the Dombrovska pit lake in June 2019. Site 1 was located in the shallow bay (depths of 20–40 cm), while sites 2, 3, and 4 were located in the other bay (depths of 1, 2.5, and 5 m, respectively) (Fig. 1). Due to the high levels of water mineralization (134 g dm^-3^) at a depth of 5 m in 2015 (Zurek et al. 2018), it was decided to collect samples only up to this depth. Communities of benthic fauna were analyzed in samples from sites 2 to 4 (at depths of 1, 2.5, and 5 m, respectively). Cytogenetical studies of *C. salinarius* were performed on larvae of the species found in the samples from sites 1 and 4. The individuals from the other two sample sites were not suitable for cytogenetic analysis.

### Physicochemical analysis of water and sediment

The samples of near-bottom water were collected using bathometers, while sediment samples were collected using an Ekman grab. In the water samples, the conductivity and pH were determined *in situ* using a WTW multimeter. The concentrations of the anions HCO_3_^−^, SO_4_^2−^, Cl^−^, and NO_3_^−^ and the cations Ca^2+^, Mg^2+^, Na^+^, and K^+^ were analyzed using ion chromatography (DIONEX ICS 1000 and IC DX 320). The water mineralization was expressed by the amount of dry residue. The dissolved oxygen was determined using the Winkler method.

For the analysis of heavy metals (Cd, Pb, Cu, Zn, Mn, and Fe) in the sediment samples, the samples were dried at 105 °C for 48 h. The silt-clay fraction (0.063 mm) was separated from each sample by sieving. Then, the sediment samples (0.5 g) were digested in Teflon tubes with a mixture of nitric (HNO_3_) and hydrochloric (HCl) acids using microwave Speed Wave, Berghof. Concentrations of all metals in the water and sediment samples were analyzed with an inductivity coupled plasma mass spectrometer (ICP-MS, Elan 6100, Perkin Elmer).

As reference data, we used data for fossil sediments (Fӧstner and Salomons 1980). The degree of contamination of the sediment at studied sites was calculated using Müller’s (1981) index of geoaccumulation (Igeo), according to the formula: *I*_geo_ = log_2_ (Cn/1.5Bn), where Cn is the measured concentration of the metal in the sediment and Bn is the geochemical background of the element in the shale (Turiekian and Wedepohl 1961). The factor of 1.5 is introduced to minimize the effect of potential local differences in the background concentration. Müller (1981) distinguished seven classes of sediment contamination; class 0, *I*_geo_ ≤ 0, uncontaminated; class 1, 0 < *I*_geo_ ≤ 1, uncontaminated to moderately contaminated; class 2, 1 < *I*_geo_ ≤2, moderately contaminated; class 3, 2 < *I*_geo_ ≤ 3, moderately to heavily contaminated; class 4, 3 < *I*_geo_ ≤ 4, heavily contaminated; class 5, 4 < *I*_geo_ ≤ 5, heavily to extremely contaminated; class 6, *I*_geo_ > 5, extremely contaminated.

### Benthic fauna

Benthic fauna from sites 2–4 were collected with an Ekman-type grab (15 × 15 cm at sites 1 and 3, and 10 × 10 cm at site 2) in replicate (× 3) to obtain quantitative samples. The samples were fixed with formalin in situ. In the laboratory, all the macro-invertebrates were picked out and their species or family were determined. The obtained material was counted per square meter.

### Cytogenetic analysis

For the cytogenetical studies, the larvae that were collected from sites 1 and 4 were fixed in alcohol:acetic acid (3:1) and then kept in the refrigerator. The number of studied individuals and salivary gland cells from both sites can be seen in Table 1. For the cytogenetic analysis, the routine aceto-orcein method was used (Michailova 1989).The chromosome preparations were done from salivary gland cells. Chromosome and larval external morphological preparations were carried out on each larva. Due to the limited content of the collected material, we used larvae of different instar: fourth instar and also the third and end of second instar. The external morphological analysis of the larvae was carried out following Schlee (1966). The mapping of the arms A, E, and F was done according to Keyl (1962), while the mapping of C and D were performed by Istomina et al. (2012) and Kiknadze et al. (2016). The chromosome arms were indicated as A1A1, B2B2, C1C1, D1D1, E1E1, F1F1, and G1G1 when the banding sequences of the arm had homozygous combinations. Sometimes the band sequences have heterozygous combinations and we have indicated these, for instance, as G1G2. Two types of chromosome rearrangements were considered: inherited, which affected all the cells of the individual, and somatic, which only occurred in a few cells of the individual. The localization of both chromosome aberrations was determined via a detailed analysis using a standard chromosome map, as done by Kiknadze et al. (2016), indicating the site of the appearance of the aberrations. All types of aberrations were calculated as percentages. To establish the percentage of inherited aberrations, the frequency of defined aberrations in all studied individuals was considered. The frequency of defined somatic aberrations was established by its appearance in the studied cells because this type of aberration occurred in only a few cells of the separate individuals.

**Table 1:** Number of studied individuals and salivary gland cells and the values for indices of *C. salinarius* from the Dombrovska pit lake

Locality	Number of individuals	Number of studied salivary gland cells	Somatic index (S)	Inherited index (*H*)
Site 1	20	552	0.60	0.15
Site 4	20	425	0.75	0.10

The somatic (*S*) and inherited indices (*H*) (Sella et al. 2004) were estimated for both sites. A somatic index was calculated for each site as the ratio of the number of different somatic aberrations relative to the number of studied individuals at that locality. The inherited index was a ratio of the number of inherited aberrations in a site to the number of the individuals studied at that site.

## Results

### Physicochemical data of the water and sediment samples

The mineralization of the near-bottom water (expressed as a dry residue) ranged between 17.3 and 26.2 g dm^−3^, the conductivity ranged between 23.5 and 35.9 mS cm^−1^, and the contents of ions (in mg dm^−3^) were as follows: Na^+^ 4023–6404, K^+^ 1166–1579, Ca^2+^ 288.4–374.4, Mg^2+^ 745.4–1036, Cl^−^ 8105–12423, SO_4_^2-^ 2949–4372, and nutrients NO_3_^−^ 7.1–15.0, and NH_4_^+^ 0.2–0.9 (Table 2). The conductivity, mineralization, and the contents of the other salinity parameters (ions Na^+^, K^+^, Cl^-^, SO_4_^2-^), and also Mg^2+^ of the near-bottom water gradually increased with increasing depth of the studied sites and were 1.4–1.5 times higher at site 4 compared with site 1. The contents of the nutrients and heavy metals in the near-bottom water had an irregular pattern. The highest concentrations of Pb and NO_3_^−^ were found at site 1, Mn and Fe at site 2, and Cd and NH_4_^+^ at site 4. The water pH was neutral (~ 7.5). The sediments at the studied sites were characterized by pH 7.0–7.5, and low concentrations of organic matter (3.3–9.2%) and heavy metals Cd, Pb, Zn, and Cu (Table 3). The concentrations of heavy metals in the reference sediment are given in Table 3 (Fӧrstner and Salomons 1980). The distribution pattern of the values of the above parameters was irregular among sites. The contents of organic matter and heavy metals in the sediments were similar at sites 1 and 4, with the exception of a lower concentration of Pb (1.6 times lower) and a higher Fe concentration (1.6 times higher) at site 4 compared with site 1. According to the *I*_geo_ values, the sediment was uncontaminated by Cu, Zn, and Pb (*I*_geo_ < 0). For Pb at site 3, the *I*_geo_ was 0.1 (slightly contaminated). The sediment was moderately contaminated (class 2) by Cd at sites 2 (*I*_geo_ = 1.9) and 3 (*I*_geo_ = 1.7) and was moderately to heavily contaminated at sites 1 (*I*_geo_ = 2.2) and 4 (*I*_geo_ = 2.1).

**Table 2:** The values of physicochemical parameters of the near-bottom water of the Dombrovska pit lake in Ukraine

Parameter	Unit	Site 1	Site 2	Site 3	Site 4
pH		7.54	7.43	7.53	7.53
Conductivity	mS cm^−1^	23.5	27.6	30.6	35.9
Na^+^	mg dm^−3^	4023	4822	5518	6404
K^+^	mg dm^−3^	1166	1255	1392	1579
Ca^2+^	mg dm^−3^	288	308	298	374
Mg^2+^	mg dm^−3^	745	836	907	1036
Cl^-^	mg dm^−3^	8105	9525	10570	12423
SO_4_^2-^	mg dm^−3^	2949	3448	3720	4372
NO_3_^-^	mg dm^−3^	11.4	15.0	7	8.9
NH_4_^+^	mg dm^−3^	0.16	0.17	0.89	0.58
Mineralization	g dm^−3^	17.3	20.2	22.4	26.2
Cd	μg dm^−3^	1.2	2.6	2.8	3.2
Pb	μg dm^−3^	3.1	3	2	1.4
Cu	μg dm^−3^	11	12	9	15
Mn	μg dm^−3^	49	867	682	429
Fe	μg dm^−3^	56	189	52	12

**Table 3:** Sediment chemistry of the Dombrovska pit lake in Ukraine

Parameter	Unit	Site 1	Site 2	Site 3	Site 4	Fossil sediment (control)*
Organic matter	%	8.9	9.2	3.3	8.4	-
pH		7.18	7.34	7.47	6.95	-
Cd	μg g^−1^	2.1	1.7	1.5	1.9	0.22
Pb	μg g^−1^	29.4	23.1	31.5	18.6	16.0
Cu	μg g^−1^	26.6	33.3	27.4	29.3	25.0
Zn	μg g^−1^	95.7	85.0	84.7	81.5	105.0
Mn	μg g^−1^	1052.0	829.7	701.4	1011.6	406.0
Fe	μg g^−1^	17366	30451	33485	27628	18200

*Förstner and Salomons (1980)

### Benthic fauna

**Fig. 2:**
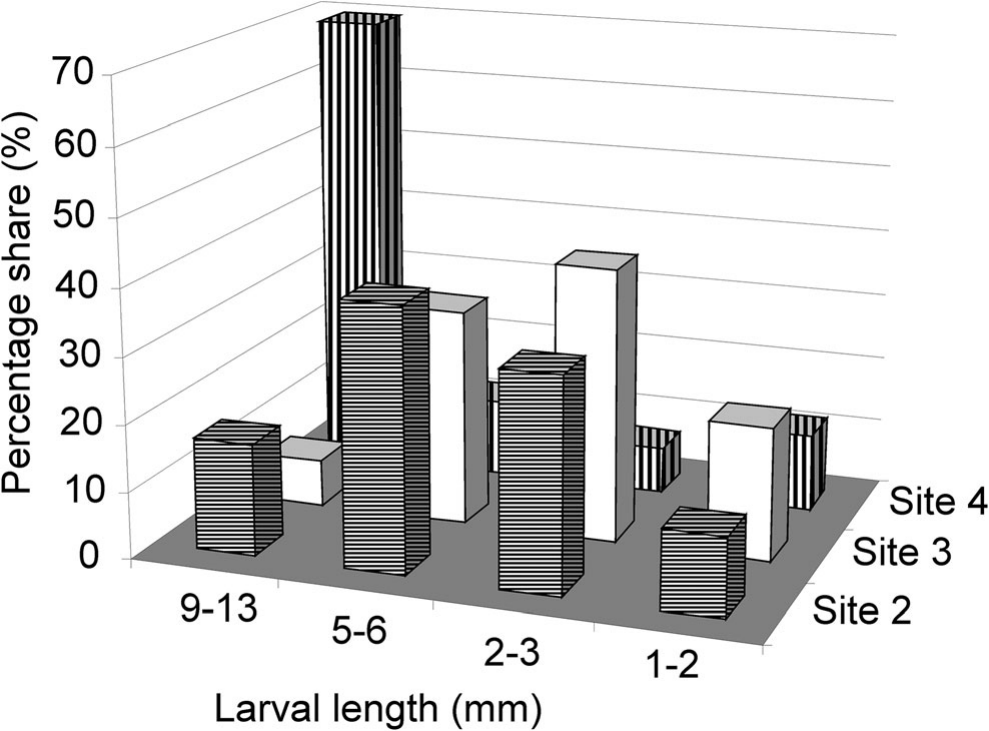
The percentage share of instar larvae (I, 1–2 mm; II, 2–3 mm; III, 5–6 mm; IV, 9–13 mm) of *Chironomus salinarius* in the Dombrovska pit lake

The density of fauna varied widely (Table 4). The highest density was found at a depth of 2.5 m (site 3), 9600 individuals/m^2^, and the lowest density was found at a depth of 5 m (site 4), 815 ind./m^2^. *Chironomus salinarius* larvae dominated at all the sites. At site 2 near the shore (a depth of 1 m), apart from *C. salinarius*, whose share was 47% of all the macro-invertebrates, there were numerous Diptera larvae from the Ceratopogonidae family (22%), Heteroptera from the genus *Sigara* (21%), and Coleoptera from the genus *Hydrotus* (7%). The share of *C. salinarius* was very high at sites 3 (85%) and 4 (78%). There, the share of the remaining groups of macro-invertebrates did not exceed 4%, except for the share of Ceratopogonidae at site 4 which was 11%. At the examined sites, all the larval instars of *C. salinarius* were found (Fig. 2). At the sites 2 and 3, the II and III instars larvae were most abundant (32–41%), while at the site 4 (a depth of 5 m) the IV instar larvae were prevalent (70%). On the surface of the water numerous exuviate of pupae of *C. salinarius* were flowing.

**Table 4:** Density (ind./m^2^) and percentage share (%) of benthic macro-invertebrate in the Dombrovska pit lake

Taxons	Site 2 (a depth of 0.5–1 m)		Site 3 (a depth of 2.5 m)		Site 4 (a depth of 5 m)	
	ind./m^2^	%	ind./m^2^	%	ind./m^2^	%
Oligochaeta–Enchytreidae	30	1	400	4	0	0
*Chironomus salinarius* Kieffer, 1915	1763	47	8167	85	637	78
*Cricotopus* (*C*) cfr. *salinophilus*	89	2	167	2	30	4
Diptera–Ceratopogonidae	815	22	200	2	89	11
Heteroptera–Corixidae–*Sigara*	800	21	367	4	30	4
Coleoptera–Ditiscidae–*Hydrotus*	252	7	300	3	30	4
Total	3748	100	9600	100	815	100

### Genome instability

**Fig 3:**
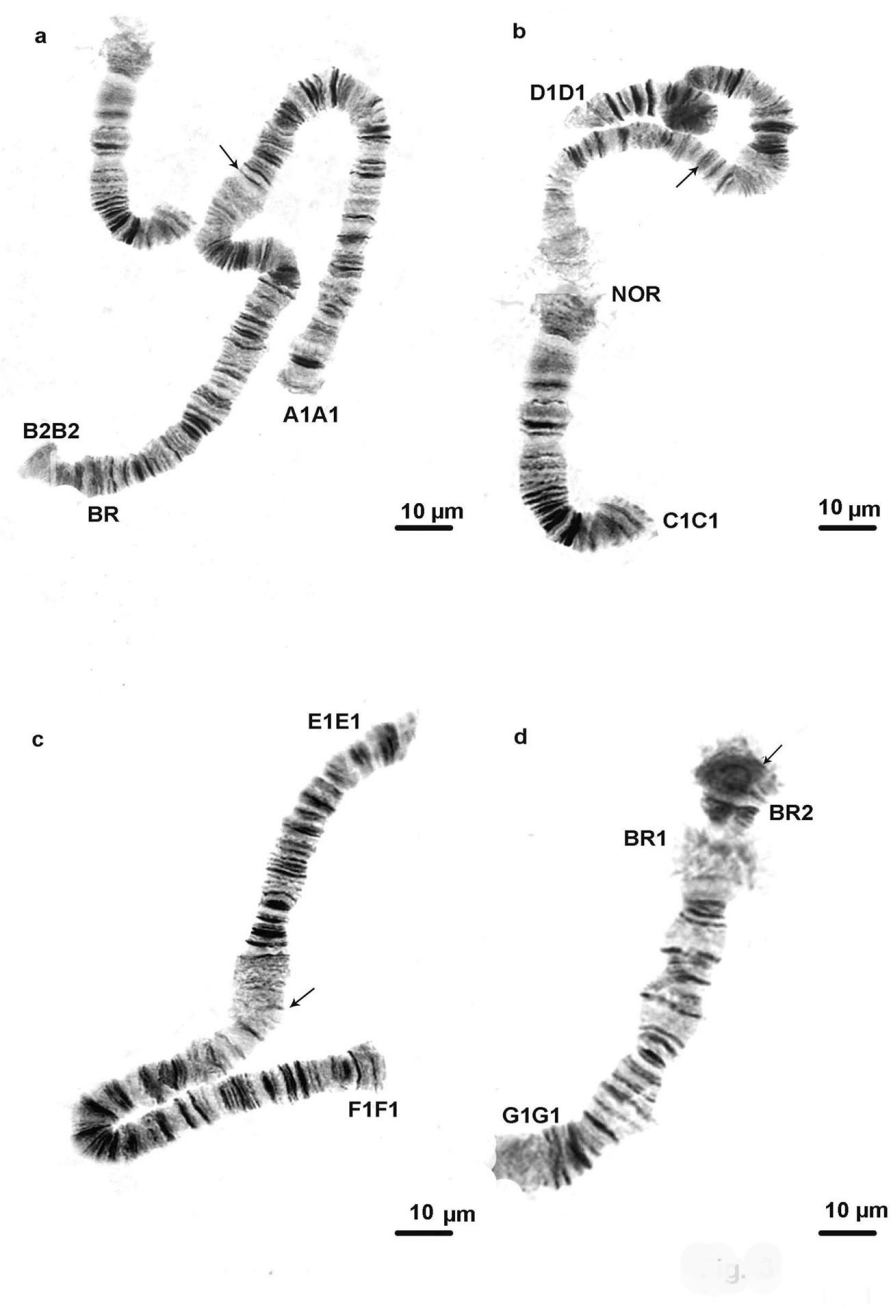
Salivary gland chromosomes in *Chironomus salinarius* from site 1 at the Dombrovska pit lake; A1A1 B2B2 chromosome (**a**), C1C1 D1D1 chromosome (**b**), E1E1 F1F1 chromosome (**c**), G1G1 chromosome (**d**). Arrow indicates the localization of the centromere region. NOR, nucleolar organizer; BR, balbiani ring

**Fig 4:**
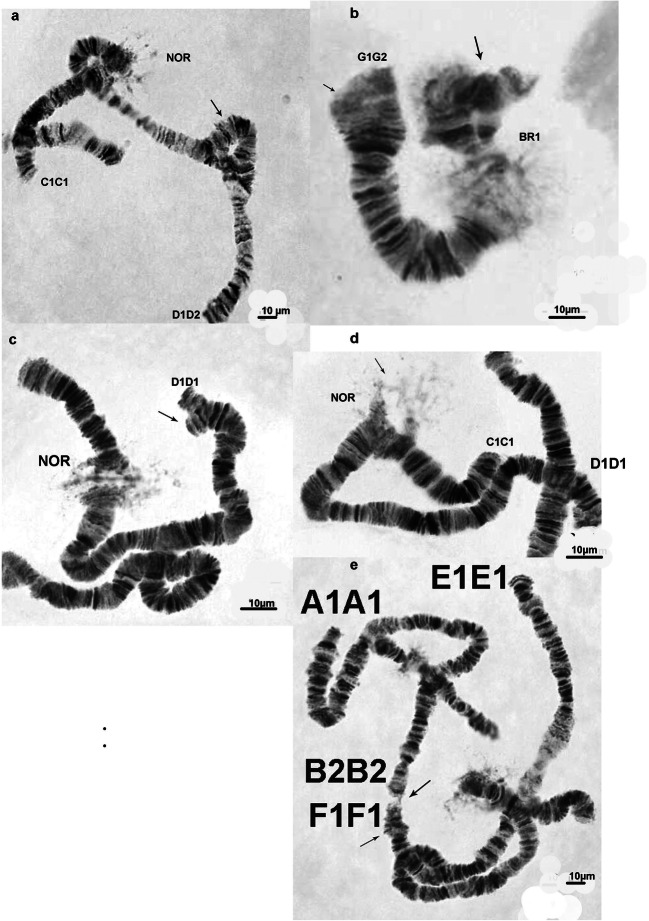
Aberrations in salivary gland chromosomes of *Chironomus salinarius* from site 1 at the Dombrovska pit lake; het. inv. in chromosome arm D1D2—sections 7–15 ***(*a**), het. inv. in chromosome G1G1–G1G2 + somatic het.inv. (**b**), deficiency in arm D1D1 (**c**), heterozygous state of NOR (**d**), ectopic contacts between chromosome arms F1F1 and B2B2 (**e**). Small arrow indicates the somatic inversion; large arrow indicates the inherited inversion

**Fig 5:**
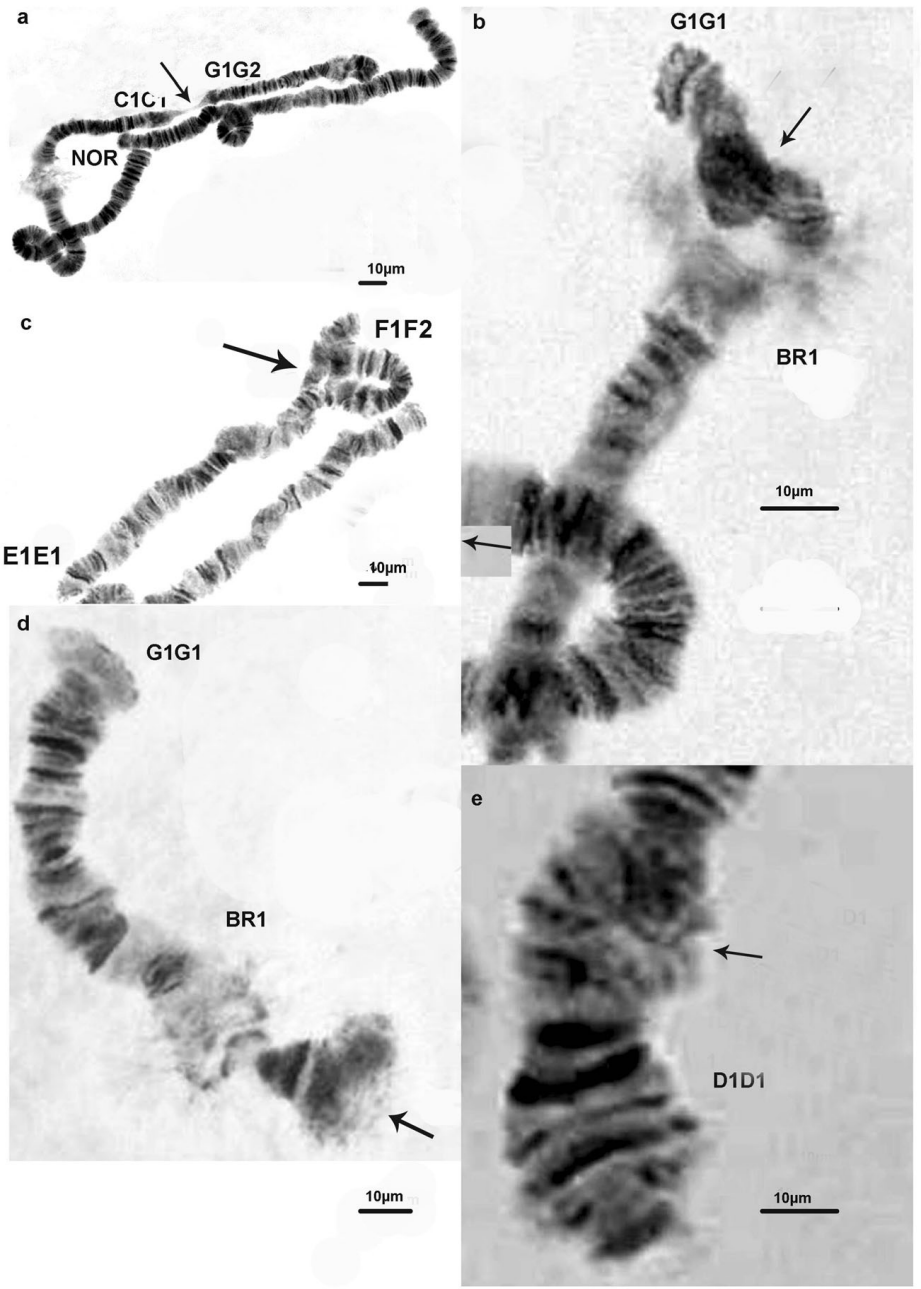
Aberrations in salivary gland chromosomes of *Chironomus salinarius* (site 1) (continue); ectopic conjugations between chromosome arms G1G2 ( heterozygous inversion in chromosome G1G1) and C1C1 (**a**), heterochromatic knob in chromosome G1G1 (**b**), het. inv. in chromosome arm F1F2—sections 14–20 (**c**), unpair part of chromosome G1G1 at centromere region; BR2 not expressed (**d**), somatic heterozygous inversion in chromosome arm D1D1 (**e**). Small arrow indicates the somatic inversion; large arrow indicates the inherited inversion

**Fig. 6:**
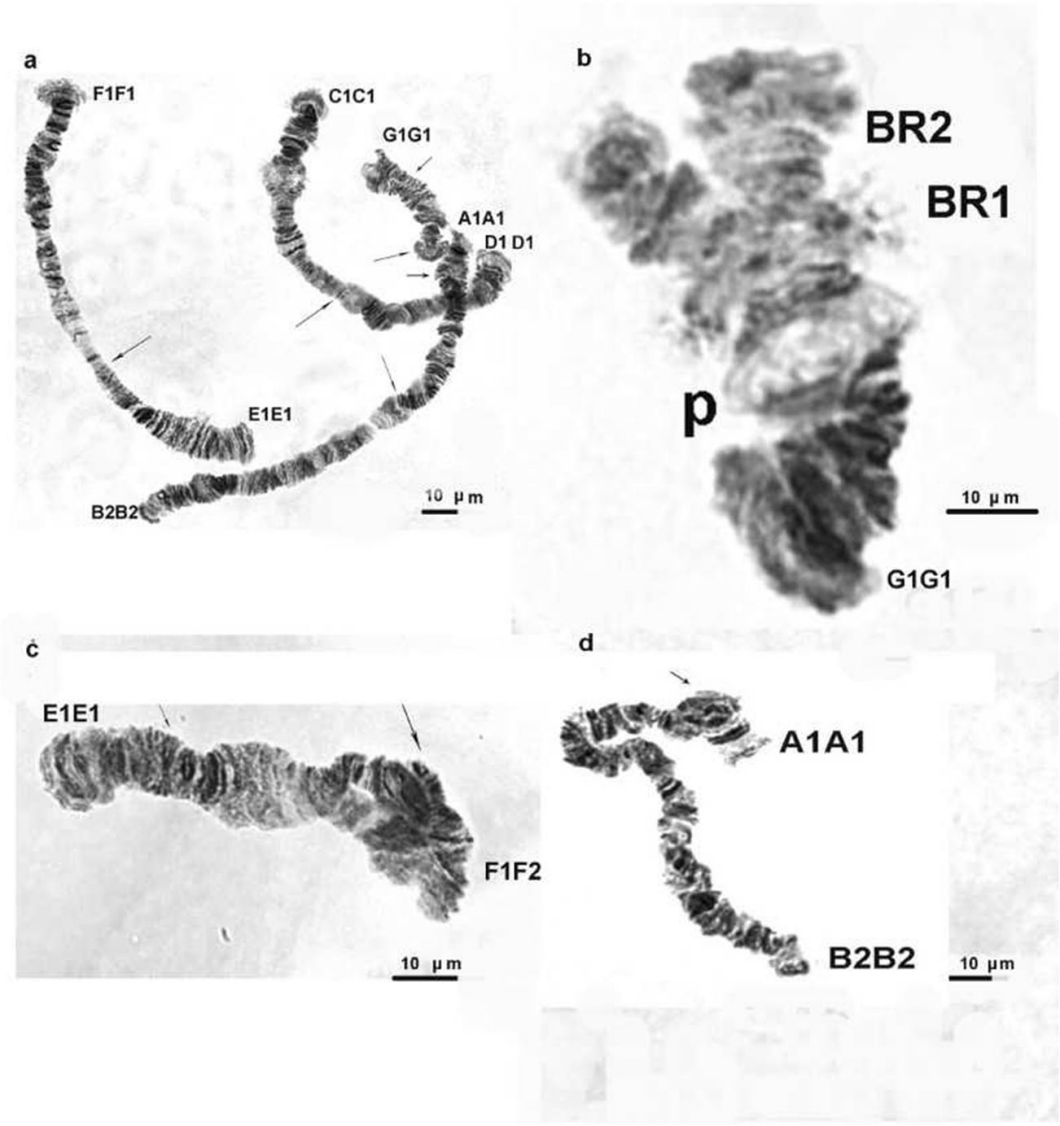
Salivary gland chromosomes and their aberrations in *Chironomus salinarius* from site 4 at the Dombrovska pit lake; polytene chromosomes A1A1 B2B2 C1C1 D1D1 E1E1 F1F1 G1G1 + somatic aberrations in arms A1A1 and G1G1 (**a**), chromosome G1G1 with a high activity of puff (p) (**b**), het. inv. in chromosome arm F1F2—sections 14–20 + somatic heterozygous inversion in arm E1E1 (**c**), somatic heterozygous inversion in arm A1A1 (**d**). Small arrow indicates the somatic inversion; large arrow indicates the inherited inversion. NOR, nucleolar organizer; BR, balbiani ring

The species from both localities (sites 1 and 4) that was studied was *C. salinarius.* The correct identification of the species was done by a detailed analysis of the salivary gland chromosomes and applying the cytogenetic markers (Kiknadze et al. 2016; Keyl 1962; Michailova 1989). Keyl (1962) considered the karyotype of the species as an unclear position. Later, according to Kiknadze et al. (2016) the species belongs to “thummi” cytocomplex with chromosome arm combinations of AB CD EF and G. The band sequences of both localities correspond to that as A1A1, B2B2, C1C1, D1D1, E1E1, F1F1, and G1G1, as described by Istomina et al. (2012), Zorina et al. (2014) and Kikinadze et al. (2016). The chromosomes A1A1 B2B2 C1C1 D1D1 are metacentric, the chromosome E1E1 F1F1 is submetacentric, and the chromosome G1G1 is telocentric (Fig. 3a, b, c, d). There is one nucleolar organizer region (NOR) in arm C1C1. In arm B2B2, there is one Balbiani ring (BR) and there are two Balbini rings (BR1 and BR2) in arm G1G1, one of them is not always expressed (Fig. 3d). The polytene chromosomes from site 1 have a distinct band-like structure (Fig. 3a, b, c, d) and those from site 4 have a disturbed structure and very often they have a granular character and some bands were fused (Fig. 6a), which made the chromosome analysis very difficult. Structural and functional changes have been established along the salivary gland chromosomes in individuals from both localities. Structural alterations are realized through somatic and inherited aberrations (Tables 5 and 6). A wide range of somatic aberrations (heterozygous inversions, deletions, deficiencies) have been identified in species from both sites (Figs. 4b, c, 5b, e, and 6a, c, d). A heterochromatic dark knob (Fig. 5b) was found in the chromosome G1G1 of the species from site 1. The somatic index (*S*) for site 1 was 0.6, while at site 4 it was 0.75 (Table 1). The established inherited aberrations in both localities (Figs. 4a, b, 5c, and 6c) made it possible to calculate the inherited index (*H*) (Table 1); for site 1, the H index was 0.15 and for site 4 it was 0.1. It is important to note that in both localities complex heterozygous inversions were found, in arms D1D1 and F1F1: D1D2 and F1F2. The inherited heterozygous inversion was observed in the chromosome arm G1G1 of individuals from the first locality (Fig. 4b). This heterozygous inversion was accompanied by asynapsis.

**Table 5:** Chromosome aberrations in *Chironomus salinarius* from site 1 at the Dombrovska pit lake

Chromosome arm	Type of aberration	Localization of the aberration	Number of salivary gland cells or individuals	Frequency in %
A	Somatic het. inv.	Pericentric het. inv.	3 cell	0.54
B	Somatic het. inv.	telomere	2 cell	0.36
C	Somatic het. inv.	Section 6	1 cell	0.18
D	Somatic deficiency	Section 1ab	1 cell	0.18
D	Inherited het.inv.	Sections 7–15	4 ind.	20
D	Somatic het. inv.	Section 11	1 cell	0.18
D	Somatic het. inv.	Section 14	1 cell	0.18
D	Somatic het. inv.	Section 18a	1 cell	0.18
F	Somatic het. inv.	Section 14	1 cell	0.18
F	Inherited het.inv.	Sections 14–20	1 ind.	5
G	Inherited het. inv.	G1/G2	5 ind.	25
G	Somatic dupl.	Between BR and BR1	1 cell	0.18
G	Somatic dupl.	Telomere	1 cell	0.18
G	Somatic deletion	Telomere	2 cells	0.36
G	Somatic het. inv.	Telomere	1 cell	0.18

*het. inv.*, heterozygous inversion; *ind.*, individual

**Table 6:** Chromosome aberrations in *Chironomus salinarius* from site 4 at the Dombrovska pit lake

Chromosome arm	Type of aberrations	Localization of the aberrations	Number of salivary gland cells or individuals	Frequency in %
A	Somatic het. inv.	Pericentric het.inv.	3 cells	0.71
A	Somatic het. inv.	Section 2e	1 cell	0.23
A	Somatic het. inv.	Section 4	2 cell	0.47
A	Somatic het. inv.	Sections 10–11	1 cell	0.23
A	Somatic het. inv.	Section 12	2 cell	0.47
B	Somatic het. inv.	In the middle	3 cell	0.71
C	Somatic het. inv.	Section 10	1 cell	0.23
D	Somatic deficiency	Section 1ab	1 cell	0.23
D	Inherited het.inv.	Sections 7–15	1 ind.	5
D	Somatic het. inv.	Section 14	2 cell	0.47
E	Somatic het. inv.	Section 2	1 cell	0.23
E	Somatic het. inv.	Sections 7–8	2 cells	0.47
E	Somatic het. inv.	Section 10b	1 cell	0.23
F	Inherited het.inv.	Sections 14–20	3 ind.	15
G	Somatic het. inv.	In the middle	1 cell	0.23
G	Somatic deletion	Telomere	2 cells	0.47
G	Somatic het. inv.	Telomere	1 cell	0.23

*het. inv.*, hetetozygous inversion; *ind.*, individual

Functional variability affects the transcriptional activity of the NOR and the Balbiani rings (BRs) in arms B2B2 and G1G1. At site 1 the NOR was in a heterozygous state ≥ 1.63% (Fig. 4d). At the same locality, in several cells of the studied individuals the BR2 in chromosome G1G1 was not expressed – 1.81%. At site 4, the NOR is not expressed or slightly expressed (7.77%). Also, BRs in arm G1G1 are slightly expressed or not expressed (7.53 %). However, it is important to underline that puff in chromosome G1G1 of individuals of this locality is well expressed in 5.18% and has a high activity in comparison with the standard (Figs. 3d and 6b).

Ectopic contacts have been observed in the polytene chromosomes of the species from both localities. At site 1, they occurred in 3.08% (Figs. 4e and 5a). They are manifested either by the binding of telomeres to different chromosomes or by binding to a small bridge between the telomeres of the following chromosome arms: G1G1 + C1C1; B2B2 + F1F1; D1D1 + B2B2; C1C1 + B2B2; B2B2 + E1E1; G1G1 + B2B2; A1A1 + G1G1; F1F1 + D1D1; E1E1 + F1F1. At site 4, they occurred between G1G1 + A1A1; D1D1 + B2B2; D1D1 + A1A1 + F1F1; D1D1 + F1F1; F1F1 + G1G1; G1G1 + B2B2; A1A1 + F1F1 in 2.12%.

## Discussion

### Biodiversity and physicochemical data of the near-bottom water and sediments

As the Dombrovska pit lake is still filling up with waters its depth is increasing, it increased by ~ 8 m between November 2015 (Zurek et al. 2018) and June 2019 (present study). In terms of salinity (17.3–26.2 g dm^−3^), the studied water of the Dombrovska pit lake may be classified as mesohaline (salinity 5–18‰) at site 1 (a depth of 20–40 cm) and polyhaline (salinity 18 do 30‰) at sites 2–4 (depths of 1, 2.5, and 5 m).

The biodiversity of benthic fauna of the pit lake was relatively small compared with other saline water ecosystems (Arias and Drake 1994; Zinchenko et al. 2019; Zorina et al. 2014), indicating the stressed conditions in the pit lake. The pit lake was inhabited mainly by species typical for saline waters: Chironomidae such as euryhaline *C. salinarius* and halophilic *Cricotopus* (C.) cfr *salinophilus*, or eurytopic (with wide ecological spectrum) taxa Coleoptera such as *Hydrotus* and Heteroptera–*Sigaria.* The larvae of *C. salinarius* (Chironomidae) dominated among the benthic fauna (47–85% of the total) which is typical for this level of salinity. This species inhabits aquatic ecosystems (rivers, lagoons, seas, and lakes) with a salinity from 6 to 80‰ (Arias and Drake 1994; Drake and Arias 1995; Gascon et al. 2007; Hiebaum 2007; Michailova 1973, 1974; Ponti et al. 2007; Zinchenko et al. 2019; Zorina et al. 2014). For example, *C. salinarius* (together with *C. salinophilus*) was extremely abundant in the rivers of the Lake Elton basin (Volgograd region, Russia) which has a salinity above 26 g dm^−3^ (Zinchenko et al. 2017; Zinchenko and Golovatyuk 2010) but does not exceed 41.1 g dm^−3^ (Zinchenko et al. 2019). In experimental studies, Cartier et al. (2011) found that for a salinity over 35 g dm^-3^ very few individuals of *C. salinarius* survive. The dominance of *C. salinarius* among benthic fauna in saline, especially hypohaline water, has also been found by other authors (Arias and Drake 1994; Gascon et al. 2007; Zinchenko et al. 2019; Zorina et al. 2014). Both the water salinity and the percentage share of *C. salinarius* (from 47 to 85%) in zoobenthos in the Dombrovska pit lake was similar to those found in the saline (13–31.9 g dm^−3^, density 49–66%) rivers of the Lake Elton basin (Volgograd region, Russia) (Zorina et al. 2014).

Apart from salinity other habitat features like loam sediments, organic matter content (3.3–9.2%), pH~7.5, a lack of macrophytes in the studied part of the Dombrovska pit lake were also typical for *C. salinarius* (Arias and Drake 1994; Zorina et al. 2014). Arias and Drake (1994) found a positive correlation between the density of *C. salinarius* and the sedimentary silt content in a lagoon fish-pond system in the Bay of Cadiz in Spain. This species has also been found in silty-sandy biotopes (Zinchenko et al. 2019). Cartier et al. (2011) indicated that food availability in the range 2–20% of organic matter does not appear to limit *C. salinarius*.

The highest density (8167 individuals/m^2^) of *C. salinarius* found at a depth of 2.5 m in the Dombrovska pit lake was lower than that found in other saline water bodies (Cartier et al. 2010; Zinchenko et al. 2019). The spatial variability of the densities of benthic fauna and *C. salinarius* is usually related to the habitat heterogeneity (Cartier et al. 2011). The lower density (1763 individuals/m^2^) of *C. salinarius* at a depth of 1 m in the Dombrovska pit lake may be associated with the pressure of predator larvae of Dytiscidae, which were numerous at the lake shore. It is more difficult to explain the drastic decrease (~ 13 times) in the density of *C. salinarius* observed at site 4 (a depth of 5 m) compared with site 3 (depth of 2.5 m) as well as the increase in the IV instar larvae at site 4 (70% of the total). The differences in the *C. salinarius* population did not clearly relate with changes in the chemical properties of the habitat. The fact that all the larval instars of *C. salinarius* were present at the same time (July 13) indicates that there are several generations during the year in the Dombrovska pit lake. This is confirmed by the occurrence of one to five generations during the year, which has been found in various water bodies (Drake and Arias 1995; Ferrarese et al. 2018; Koskinen 1968). According to Cartier (2011), the time of development increased with an increase in the salinity levels.

### Genome instability

In both localities, the standard karyotype is predominant. The population that was studied differed from the German (Keyl 1962) and Bulgarian (Michailova 1974) populations by homozygous inversions in arm B-B2B2, which is predominant at both sites of the Dombrovska pit lake. The same homozygous inversion was fixed in the Chernovska and Pantsug (Russia) population (Zorina et al. 2014). The *C. salinarius* of the two studied localities of the Dombrovska pit lake differed in the range and frequency of inherited rearrangements in comparison with a Black Sea population in Bulgaria (Michailova 1973, 1974), Ukraine (Grinchuk 1979, 1984), and some Russian populations (Kiknadze et al. 2016; Zorina et al. 2014). Arms A1A1 and C1C1 are monomorphic in the studied localities concerning the inherited inversions, while arm A is polymorphic in the Black Sea population (Michailova 1973). Arms D1D1 and F1F1 are polymorphic in the studied materials, similar to Bulgaria (Michailova 1973) and some Ukraine (Grinchuk 1984) populations, respectively. Also, arm G1G1 is polymorphic as it is in some Russian populations (Zorina et al. 2014; Kiknadze et al. 2016). In all these populations a complex heterozygous inversion was established. In our study, the heterozygous inversion G1G2 (Fig. 4b) provoked the asynapsis. There were different reasons for asynapsis: chromosome rearrangements, hybrid origin, some internal physiological factors, different point mutations, and the heterocyclicity of the paternal and maternal chromosomes can all elucidate the causes of asynapsis (Zhimulev, 1996).

However, the aberrations occurred at different frequencies. As White (1977) underlined, the chromosome polymorphism is adaptive in certain conditions. Both environmental and geographic gradients have been defined and correlated with variations in the different types and frequency of aberrations which provide the adaptive potential of the species (King 1993). In all populations of the species that have been cytogenetically studied so far, a large variation in the salinity of the waters inhabited by the species has been observed. For instance, the salinity in Bulgarian populations (Pomoriisko lake) varies between **S** = 42‰ in June and *S* = 60‰ in December (Hiebaum 2007). Also, a variation in salinity was found in Russian populations (Zorina et al. 2014). A more stressful condition probably exists at site 4, which influences the polytene chromosomes of *C. salinarius*: the chromosomes become much shorter, and many of the easily recognized neighboring bands fuse to form blocks of bands. Changes in the appearance of the polytene chromosomes have been observed in the *Chronomus valkanovi* (Michailova 1973, 1974) that occur in biotopes with extremely high salinity as high as 260‰.

Another important response of the *C. salinarius* genome was the somatic aberrations in the polytene chromosomes of the species, which is usually induced by the heavy metals deposited in the sediments, found in other species (Ilkova et al. 2018; Michailova et al. 2018). It is important to underline that somatic aberrations were not found in other populations of the species (Michailova 1973; Zorina et al. 2014; Kiknadze et al. 2016). These aberrations were established at this study for the first time. The Cd concentrations suggest that the sediment is moderately contaminated. The Cd concentrations were also elevated ~ 10 times higher than the control (Table 3) (Förstner and Salomons 1980). However, the concentrations of metals both in the water (Cd, Pb, and Cu) and sediment (Cd, Pb, Cu, Zn) of the Dombrovska pit lake were similar to those found in small contaminated water bodies (Szarek-Gwiazda et al. 2018). The metal concentrations in sediment were below the threshold concentrations of probable effect level (PEL, Cd 3.53 μg g^−1^, Pb 91.3 μg g^−1^, Cu 197 μg g^-1^, Zn 315 μg g^-1^) (Smith et al. 1996) above which adverse effect of metals on the organisms are expected to occur frequently.

The larval material was collected at the same time as the sediment and water were taken for analysis. The larvae were exposed to the fluctuation of the salinity level of the lake during their development. The lake conditions influenced the appearance of many somatic aberrations and changes in the functional activity of Balbiani rins and Nucleolar Organizer Region, whose appearance was provoked by the specific environmental conditions.

Despite low concentrations of the heavy metals, it is possible that the interactions between the heavy metals and the complexes that are formed may induce somatic changes. Such a phenomenon has been observed in other species (Baršienė 2003). Also, it is quite possible that the physicochemical conditions, the continuous mixing of saline and freshwater leading to a decrease in the salinity and an increase in the water level of the pit lake induce a specific response in the species genome.

Along with the structural chromosomal changes in the genome of *C. salinarius*, we found changes in how it functions. These changes affect key structures in the genome: the NOR and the BRs. The BRs are very important structures as they are sites of intensive transcription of genes encoding for silk proteins (Wieslander 1994). The silk proteins are used by Chironomids in the construction of the tube in which the larvae live and develop. Both BR1 and BR2 in *C. salinarius* are a species specific sign and can be seen in at all instar of the larval development, where both BRs show different activity. However, larvae at different instars were examined, this did not allow us to make a detailed statistical analysis of the functional activity of the Balbiani rings, as has been done in the other Chironomid species under stress conditions (Beermann 1973). Nevertheless, BR2 on chromosome G1G1 was not expressed in 8 cells from a total of 425 cells (1.88%), examined from site 4. It is quite possible this effect was caused by the physicochemical parameters of the lake where the larvae lived and developed. Furthermore, the depression of the functional activity of one of the BRs was observed in the experimental exposure of *C. riparius* to specific trace metals (Michailova et al. 2012; Planello et al. 2007).

Future molecular genetic studies and laboratory studies of *C. salinarius* will hopefully shed light on this process. In addition, future research under experimental conditions could show the relationship between certain types of aberration and the concentration of some trace metals.

## Conclusions

Studies were carried out in the western part (up to a depth of 5 m) of the Dombrovska pit lake in Ukraine, which formed in a former potassium salt mine that was filled with brine and freshwater. This process has caused the salinity to decrease in the upper layer and the water level to increase. The diversity of the benthic fauna was poor and dominated by *C. salinarius*. The habitat parameters, such as the mesohaline and polyhaline water (17.3–26.2 g dm^−3^), loamy sediment, pH, and content of organic matter, are all suitable for this species. The other taxa, Hetereoptera and Coleoptara, were only more numerous in the coastal zone (a depth of 0.5–1 m). The observed ectopic pairing between chromosomes, together with alterations in the structure of the polytene chromosomes and changes in the functions of the key structures (NOR and BRs) of the polytene chromosomes could be due to the specific living conditions, i.e., continuous mixing of saline and freshwater resulting in a decrease in salinity and an increase in the water level.
